# Paeoniflorin Derivative in Paeoniae Radix Aqueous Extract Suppresses Alpha-Toxin of *Staphylococcus aureus*

**DOI:** 10.3389/fmicb.2021.649390

**Published:** 2021-03-20

**Authors:** Xiaoye Liu, Yafei Zhang, Zengshun Li, Pengpeng Zhang, Ying-Jian Sun, Yi-Jun Wu

**Affiliations:** ^1^Beijing Key Laboratory of Traditional Chinese Veterinary Medicine, Department of Veterinary Medicine and Animal Science, Beijing University of Agriculture, Beijing, China; ^2^Laboratory of Molecular Toxicology, State Key Laboratory of Integrated Management of Pest Insects and Rodents, Institute of Zoology, Chinese Academy of Sciences, Beijing, China; ^3^Department of Mechanics and Engineering Science, College of Engineering, Peking University, Beijing, China; ^4^National Center for Veterinary Drug Safety Evaluation, College of Veterinary Medicine, China Agricultural University, Beijing, China

**Keywords:** Paeoniae Radix aqueous, bacterial infection, antitoxin, *Staphylococcus aureus*, alpha-toxin

## Abstract

The emergence and dissemination of bacterial infections is paralyzing our public health systems worldwide. Worse still, there are no effective antibiotics against bacterial toxins, which facilitate the infection. Natural herbs that target bacterial toxins may be a better choice for therapy of infectious diseases. However, most natural drugs present unknown compositions and unclear mechanisms. Here we demonstrated that the Chinese herb Paeoniae Radix aqueous extract (PRAE) could suppress alpha-toxin (α-toxin) of *Staphylococcus aureus*. We observed that the paeoniflorin derivative (PRAE-a) derivative in PRAE significantly abolished the hemolytic activity of *S. aureus* α-toxin. The analyses of high-performance liquid chromatography (HPLC), mass spectrometer (MS), Fourier transform infrared spectrometer (FTIR), and nuclear magnetic resonance (NMR) showed that PRAE-a was a glycoside compound with a paeoniflorin nucleus. We further found that PRAE-a disrupted the pore-forming ability of α-toxin by prevention of the dimer to heptamer. Therefore, PRAE-a proved to be an effective therapy for *S. aureus* lung infections in mice by inhibiting α-toxin. Collectively, these results highlighted that PRAE-a can be used as an antibacterial agent to attenuate *S. aureus* virulence by targeting α-toxin.

## Introduction

Antibiotics are the most effective antimicrobial drugs that protect humans and animals against infectious disease. However, the factors of antibiotic resistance, disturbance of intestinal flora, and the disruption of host microenvironment caused by antibiotic therapy are holding back the continued use of antibiotics (Sommer et al., 2017; Stapels et al., 2018). Antibiotic resistance especially is a global threat for both human and animal health (Bonten, 2016; Meylan et al., 2018; Bakkeren et al., 2019). In addition, the discovery and development of new antibiotics is quite limited due to the rapid emergence of resistance (Brown, 2015). In addition, antibiotic residue has large uncertain risk. For instance, antibiotics can destroy the gut microbiota to drive an expansion of pathogenic bacteria and lead to re-infection (Rivera-Chavez et al., 2016; Liu et al., 2020a). Reports showed that tetracyclines disturbed mitochondrial function in various eukaryotic cells (Moullan et al., 2015). Beyond that, antibiotics can increase the production of bacterial toxins (Angel Villegas et al., 2015) and further facilitate bacterial infections (Vadia et al., 2011; Maurer et al., 2015; Cohen et al., 2016). However, at present, there are few drugs for clearance of bacterial toxins. Thus, discovery of new antitoxin drugs and illustration of the action mechanism are urgent.

Although the antibody-antibiotic conjugates (AAC) drugs that targeted the *S. aureus* α-toxin were developed to eliminate the intracellular *Staphylococcus aureus*, antibiotics are still used in these new drugs (Lehar et al., 2015; Mariathasan and Tan, 2017). Therefore, more efforts are needed to develop antitoxin drugs in natural drugs. There are many components in natural herbs that show antibacterial or antitoxin activity (Dutkiewicz et al., 2001; Li et al., 2019; Song et al., 2020a). Since *S. aureus* α-toxin can even cause the infection of Gram-negative bacteria such as *Pseudomonas aeruginosa* and *Klebsiella pneumoniae* (Cohen et al., 2016), the drug discovery for antitoxins is worth researching. Although the activities of the direct antibacterial activation in natural herbs are mostly weaker than that of antibiotics (Si et al., 2016; Quinn et al., 2017), the multi-target mechanisms of the antibacterial effect of herbs were exhibited and should be further explored (Si et al., 2016; Li et al., 2019; Lin et al., 2020).

In this work, we aimed to investigate the antibacterial function of Radix Paeoniae Rubra, a kind of Chinese herb, which derives from red peony root and belongs to the dried root of the ranunculaceae plant *Paeonia lactiflora* (Shi et al., 2016). Based on the theory of traditional Chinese medicine, Radix Paeoniae Rubra has the effects of clearing heat, cooling blood, promoting blood circulation, and removing blood stasis (Yan et al., 2018). Our results showed that Paeoniae Rubra aqueous extract inhibited the hemolytic activity of *S. aureus* α-toxin *in vivo* and *in vitro*. The main component of the antihemolytic activity in the extract is termed as PRAE-a, which has a paeoniflorin - like structure. PRAE-a abolished α-toxin by increasing dimer accumulation. Finally, animal model experiments also showed that PRAE-a has a therapeutic effect against *S. aureus* lung infections by suppression of α-toxin *in vivo*.

## Materials And Methods

### Preparation of Paeoniae Radix Aqueous Extract

Radix Paeoniae Rubra were purchased from Beijing Tonrentang Traditional Chinese Herbs Co. Ltd. The standard chemicals albiflorin, benzoylpaeoniflorin, oxypaeoniflorin, paeonol, and paeoniflorin were purchased from J&K Chemical Co. Ltd. Radix Paeoniae Rubra were soaked in DI water for 30 min before use. Then the slices were heated at 100°C and boiled until boiling and boiled for another 30 min. This process was repeated to obtain the decoction of Paeoniae Radix aqueous extract. The powder of the extract was obtained by vacuum drying as previously reported (Liu et al., 2020a). The main chemical constituents of Radix Paeoniae Rubra, including albiflorin, benzoylpaeoniflorin, oxypaeoniflorin, paeonol, and paeoniflorin, were all purchased from Beijing J&K Scientific Ltd.

### Hemolytic Activity Assay

*S. aureus* α-toxin (Sigma-Aldrich, 50 μg/ml) or the supernatants of *S. aureus* ATCC29213 (1 × 10^9^ CFU/ml, collection from 6 h growth bacteria) were incubated with compounds for about 1 h. Then mixtures of samples were added into 5% fresh rabbit blood cells for 30 min to detect the hemolytic activity. The positive control of anti-hemolytic activity of α-toxin used was MED14983^*^ (Sigma-Aldrich, 5 μg/ml). The hemolysis rates of compounds were detected by a plate reader (Tecan Infinite 200pro) and calculated based on previous protocol (Liu et al., 2017, 2020d) as below,

Hemolysis rate (%) = [OD_450 sample_ – OD_630 nm blank_] / [OD_450 0.1% Triton X-100_ – OD_630 nm blank_] × 100%

### High Performance Liquid Chromatography

C18 column, Inertsil ODS-3 (4.6 × 250 mm, Particle size 5 μm); Mobile phase:water (A)-acetonitrile (B), Gradient elution procedure: 0–10 min, 95% A, 10–20 min, 95-90% A, 20–30 min, 90-80% A, 30–35 min, 80–100% A. Flow rate: 1 ml/min, UV detector, Detection wavelength: 230 nm; Injection volume 50 μ; Column temperature 25°C.

### Mass Spectrometer

High performance liquid chromatography mobile phase methanol (97%), mobile phase flow rate: 0.8 ml/min; Sample dosage: 10 L; Detection wavelength: 270 nm; Column temperature: 30°C. Electrospray mass spectrometry (ESMS) was performed using a positive and negative ion mode. Mass charge ratio (M/Z) detection range: 50~1000; Spray voltage: 4500 V, sheath gas and auxiliary gas are nitrogen, metal capillary temperature: 260°C, metal capillary voltage 20 V.

### Fourier Transform Infrared Spectrometer Assay

The analysis of FTIR were carried out by Shanghai WEIPU Chemical Technology Service.^1^

### Nuclear Magnetic Resonance Analysis

Structure Analysis involved drying the extracts and sending them to Shanghai Microspectral Analysis and Testing Center for Fourier Transform Infrared Spectrometer (FTIR; United States Thermo Scientific Nicolet IS5) detection and Nuclear Magnetic Resonance (NMR) analysis (German Bruker Avance III400MHZ).

### Native Polyacrylamide Gel Electrophoresis

*S. aureus* α-toxin (50 μg/ml) was treated with PRAE at the concentrations of 0.06, 0.15, 0.25, 0.5, 1, and 2 mg/ml for 30 min. Sodium deoxycholate (NaDC, 5 mM) was used as the control to prevent α-toxin dimerization. PRAE (0.15, 0.5, and 2 mg/ml) interacted with α-toxin and was treated with or without NaDC for 30 min. Then the proteins were separated by native polyacrylamide gel electrophoresis with 8% polyacrylamide gels without β-mercaptoethanol and DDT. Finally, the bands were identified by silver staining method.

### Ethics Statement

Mice (five-weeks-old) were purchased from the Academy of Military Medical Sciences, Beijing, China (Certificate Number: SCXK-PLA 2012-0004). All animal experiments were approved by the Institutional Animal Care and Use Committee at the Academy of Military Medical Sciences Institute (Beijing, China; approval no. SYXK2014-0002).

### Mouse Infection and Treatment

4-week-old female ICR mice (~30 g) were randomly divided into four groups (n ≥ 10 every group). Intranasal infection of *S. aureus* (10^9^ CFU/g) were the infected groups. No infected mice served as the negative control. The mice treated by MED14893* and α-toxin were used as positive control. PRAE-a (1 mg/g) was treated with *S. aureus* infected mice. Mice were also treated with *S. aureus* α-toxin (50 ng/g). After infection and treatment, the mouse lung area was determined and recorded and the lung tissues of the mice were collected to detect the concentrations of α-toxin by ELISA. The numbers of *S. aureus* were counted by colony count technique (CFUs), according to previously published methods (Liu et al., 2020b,c). All mice both dead and alive were used for analysis.

### Statistical Analysis

Statistical analysis was performed using GraphPad Prism 8.0 software. Results were expressed as means ± SD and *p*-values were calculated using unpaired *t*-test between two groups or one-way ANOVA among multiple groups. All mice were used for analysis. All experiments were performed at least in triplicate.

## Results

### PRAE-a in Paeoniae Radix Aqueous Extract Had Significant Antihemolytic Activity

**Figure 1:**
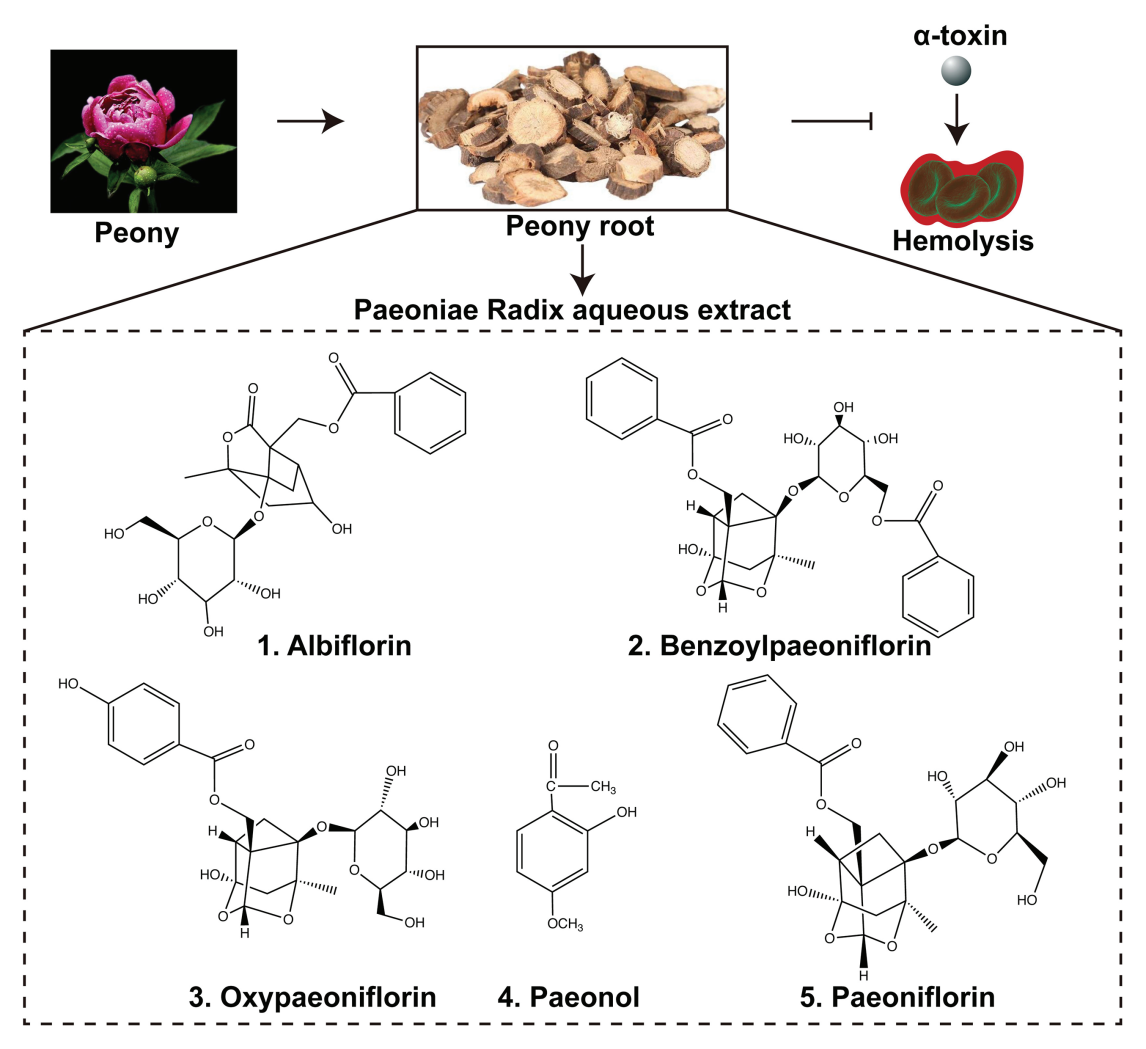
Paeoniae Radix aqueous extract and the active ingredients. Paeoniae Radix aqueous extract (PRAE) were prepared from the traditional Chinese herb Peony root. The extract could interrupt the hemolysis of α-toxin. There are five main active ingredients in the extract: albiflorin, benzoylpaeoniflorin, oxypaeoniflorin, paeonol, and paeoniflorin. The chemical structures of the five ingredients were illustrated in the Figure.

**Figure 2:**
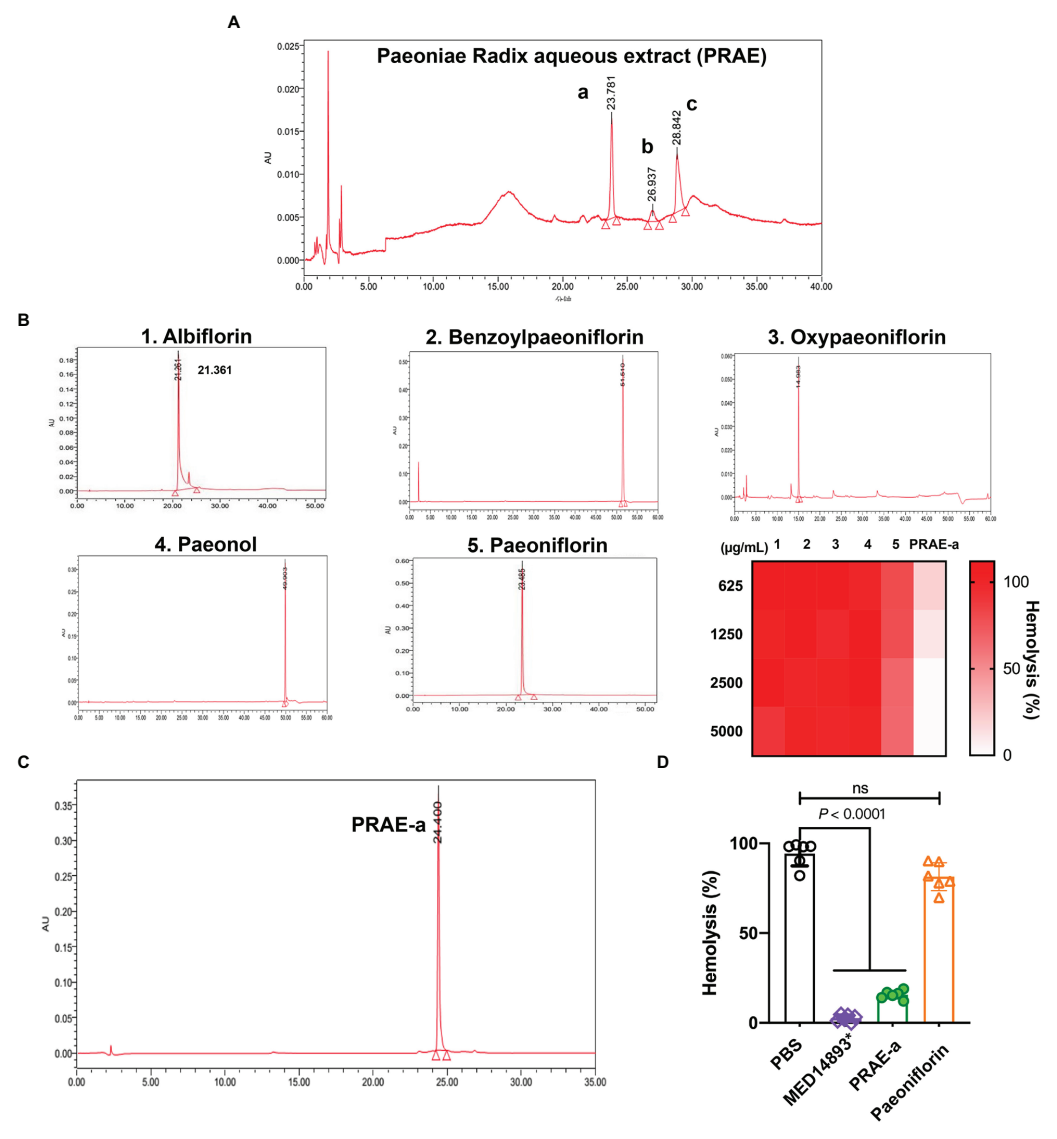
The antihemolytic activity of the main component in the Paeoniae Radix aqueous extract. **(A)** PRAE-a, PRAE-b, and PRAE-c were separated from the Paeoniae Radix aqueous extract at the peak times of 24.773, 36.832, and 45.423, respectively. **(B)** The standard ingredients of the extract, which include albiflorin, benzoylpaeoniflorin, oxypaeoniflorin, paeonol, and paeoniflorin, were detected by HPLC. **(C)** PRAE-a showed a higher antihemolytic activity than the standard drugs against α-toxin. **(D)** PRAE-a effectively inhibited the hemolytic activity of α-toxin from *S. aureus*. The supernatants of *S. aureus* were incubated with PBS, MED14893^*^, PRAE-a, or paeoniflorin for 1 h to detect the hemolytic activity. “ns” indicated no significance (*p* > 0.05). Data were shown as Mean ± SD (*n* = 6).

The main active ingredients of the Paeoniae Radix extract include albiflorin, benzoylpaeoniflorin, oxypaeoniflorin, paeonol, and paeoniflorin (Figure 1). We found that Paeoniae Radix aqueous extract (PRAE) could inhibit the hemolytic function of bacterial toxins. However, the specific antitoxic mechanisms of PRAE and the structure of active components in the extract are not clear. Thus, to analyze the compounds in PRAE, HPLC was employed to separate the extract. Results showed that there are three main peaks in the sample HPLC spectrum and we named them PRAE-a (24.773 min), PRAE-b (36.832 min), and PRAE-c (45.423 min; Figure 2A). The retention time of PRAE-a was similar to that of paeoniflorin (23. 5 min), while the retention time of PRAE-b and PRAE-c was different from that of other chemical components as demonstrated by the fact that the peak times of hydroxypaeoniflorin, paeonol, albiflorin, and benzoylpaeoniflorin were at 14.0, 51.5, 21.3, and 49.9 min, respectively, (Figure 2A). We further discovered that PRAE-a might be paeoniflorin by comparing the standard drugs of active ingredients in the extract, which include albiflorin, benzoylpaeoniflorin, oxypaeoniflorin, paeonol, and paeoniflorin, because PRAE-a and paeoniflorin had the closest peak times at 24.773 and 23.485, respectively (Figures 2A,B). And we further discovered that PRAE-a had the best antihemolytic activity of α-toxin compared to these standard drugs (Figure 2B). To further investigate the antihemolytic function of PRAE-a, we prepared plenty of PRAE-a from original drugs (Figure 2C). We employed specific neutralizing antibodies of α-toxin (MED14893^*^) as the positive control and used supernatants of *S. aureus* to detect the antihemolytic activity of PRAE-a. Results showed that PRAE-a have similar antihemolytic activity effects as MED14893^*^ (Figure 2D).

### PRAE-a Is a Glycoside Compound With a Paeoniflorin Nucleus

**Figure 3:**
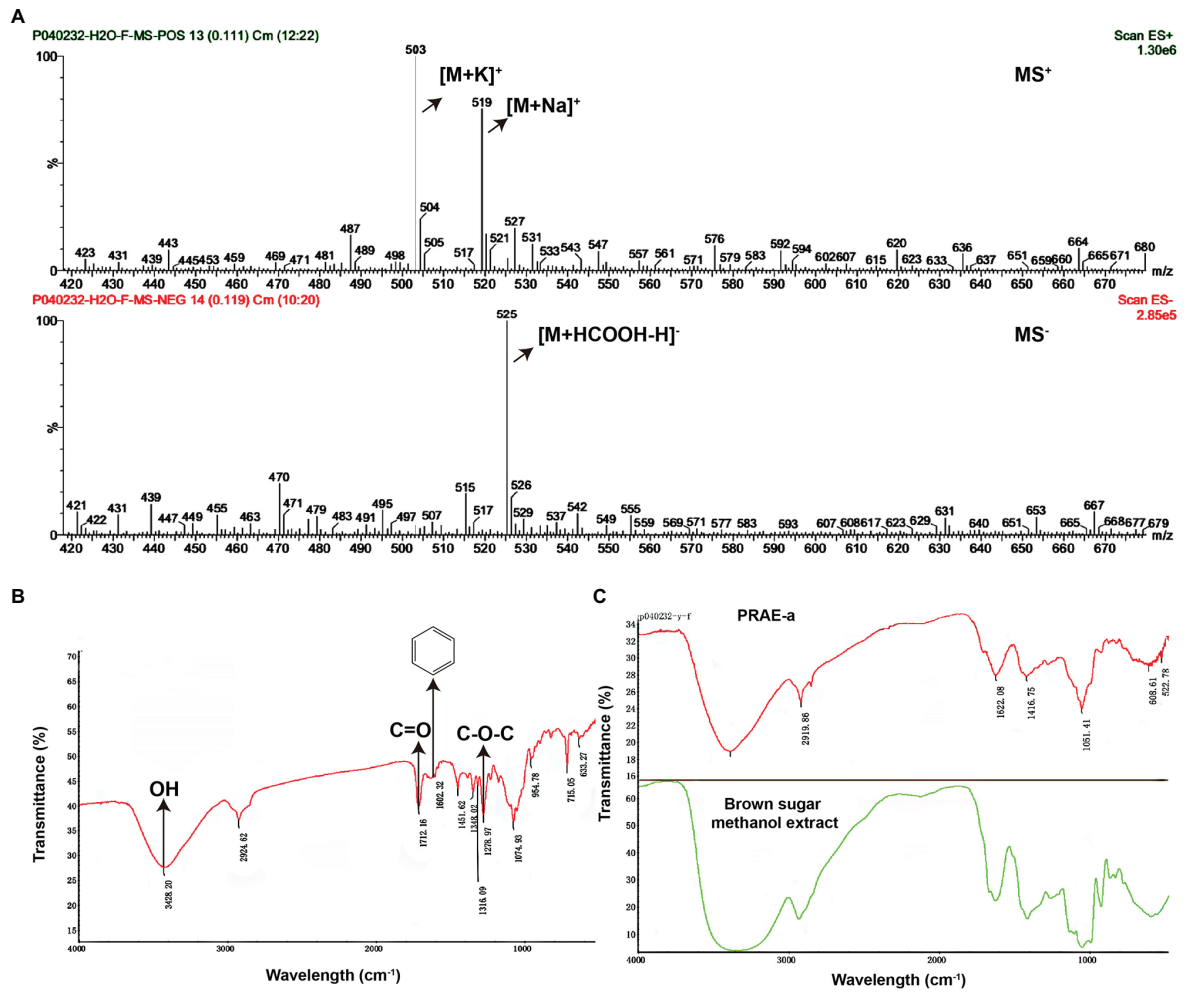
Mass spectrometry and Fourier transform infrared spectrometer analysis of the structures in the extract compounds. **(A)** Mass spectrometry (MS) analysis showed that there is a chemical in the extract which has a paeoniflorin-like structure. The MS^+^ (upper) showed that the peaks of [M + Na]^+^ are at m/z = 503 and [M + K]^+^ at m/z = 519. The MS^−^ (bottom) showed the peak of [M + HCOOH-H]^−^ is at m/z = 525. **(B)** Fourier transform infrared spectrometer (FTIR) determination indicated that PRAE-a, a main component in the extract, is similar in structure to paeoniflorin. The signals that represent hydroxy, carbonyl group, benzene ring, and ether bond appeared, respectively, at the positions of wavelengths of 3428, 1712, 1602.32, and 1279 cm^−1^, which matched with the observations at the wavelengths for the assay of the chemical paeoniflorin. **(C)** The extract had the structure of a carbohydrate. The brown sugar methanol extract (green line) had a similar formation in the wavelength to the extract (red line).

**Figure 4:**
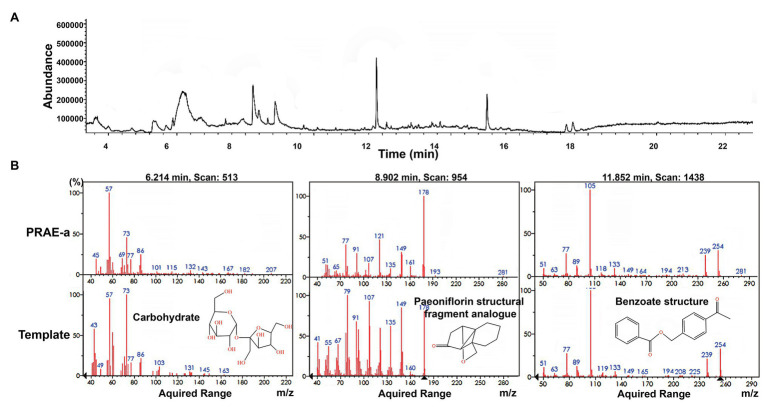
Gas chromatography mass spectrometry (GS-MS) analysis of PRAE-a. **(A)** The whole GC-MS diagram of the extract. **(B)** The comparison with template structure showed that there existed carbohydrate, paeoniflorin structural fragment analogue, and benzoate structure in the extract.

**Figure 5:**
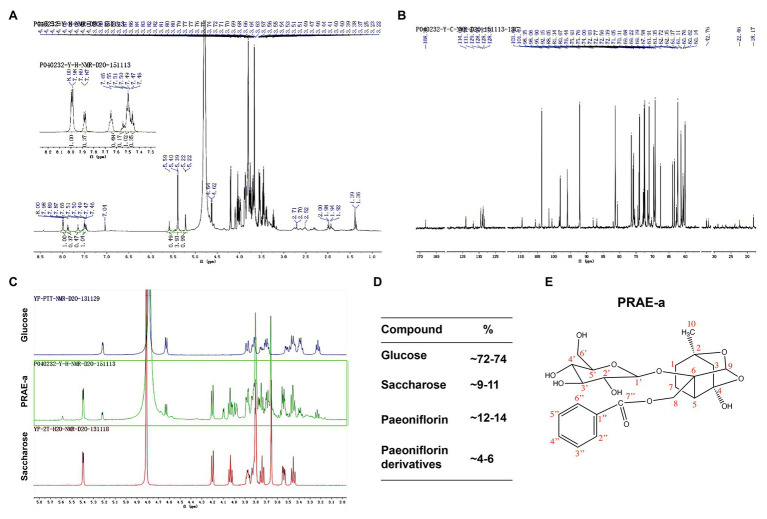
Nuclear magnetic resonance (NMR) analysis of the extract compounds. **(A)** The ^1^H-NMR image of the extract. The upper left corner is a partial magnification of the aromatic ring region. **(B)** The ^13^C-NMR image of PRAE-a. **(C)** Comparison of glucose or saccharose with the extract by ^1^H-NMR analysis. **(D)** The proportion of glucose, saccharose, paeoniflorin, and paeoniflorin derivatives were approximately 72-74%, 9-11%, 12-14%, and 4-6%, respectively. **(E)** The structure of paeoniflorin and its carbon sequence (red) in the extract.

To further characterize the structure of PRAE-a, mass spectrometry (MS) analysis was used to detect the functional groups of the component. MS images showed that PRAE-a is similar to the structure of paeoniflorin (Figure 3A), as shown in the peaks of [M + Na]^+^ at m/z = 503 and [M + K]^+^ at m/z = 519 from MS^+^ image (upper) and the peak of [M+HCOOH-H]^−^ at m/z = 525 from MS^−^ image (bottom). Meanwhile, Fourier transform infrared spectrometer (FTIR) analysis showed that PRAE-a had a paeoniflorin-like structure through detecting the hydroxy, carbonyl group, benzene ring, and ether bond at the wavelength of 3428, 1712, 1602.32, and 1279 cm^−1^, respectively (Figure 3B). Interestingly, we found that PRAE might contain a sugar structure due to the similar waveform to brown sugar methanol extract (Figure 3C). Moreover, gas chromatography mass spectrometry (GC-MS) analysis indicated that the structure of benzoate and the structural fragment of paeoniflorin might be derived from PRAE-a, while the carbohydrate might be derived from sucrose and glucose (Figure 4). The analysis of PRAE-a by ^1^H- and ^13^C-nuclear magnetic resonance (NMR) showed that there existed sugar components in PRAE-a (Figures 5A,B). Subsequently, the comparison of the ^1^H-NMR spectrum between glucose and saccharose or PRAE-a showed that the proportion of glucose, saccharose, paeoniflorin, and paeoniflorin derivatives were approximately 72-74%, 9-11%, 12-14%, and 4-6%, respectively (Figures 5C,D and Table 1). It was exhibited that PRAE-a had a paeoniflorin-like structure and had a different carbon sequence (red) from paeoniflorin (Figure 5E). Altogether, the results illustrated that PRAE-a had a paeoniflorin-like structure and it could be deduced that the carbohydrate from PRAE-a was necessary.

**Table 1:** The data analysis of ^1^H-NMR and ^13^C-NMR in PRAE-A.

Position of carbon atoms	^1^H-NMR	Reference	^13^C-NMR	Reference
1	/	/	88.0	89.3
2	/	/	86.9	87.2
3	1.98, 2.32	1.81, 2.19	42.8	44.5
4	/	/	105.6	106.4
5	2.52	2.58	42.2	43.9
6	/	/	72.9	72.2
7	1.98, 2.70	1.95, 249	22.5	23.4
8	4.79	4.74	61.1	61.7
9	5.59	5.41	101.5	102.2
10	1.39	1.36	18.2	19.6
Glc-1’	4.58	4.52	100.6	100.1
2’	3.17-3.35	3.17-3.33	75.4	75.0
3’	3.17-3.35	3.17-3.33	80.7	78.0
4’	3.17-3.35	3.17-3.33	72.9	71.7
5’	3.17-3.35	3.17-3.33	80.7	77.9
6’	3.57, 3.84	3.59, 3.84	62.7	62.9
Ben-1”	/	/	131.7	131.2
2”	8.00	8.06	129.5	130.7
3”	7.50	7.48	128.8	129.6
4”	7.65	7.61	134.0	134.4
5”	7.50	7.48	128.8	129.6
6”	8.00	8.06	129.5	130.7
7”	/	/	168.3	168.0

### PRAE-a Destroyed the Hemolytic Activity of α-Toxin by Preventing the Dimer to Heptamer

**Figure 6:**
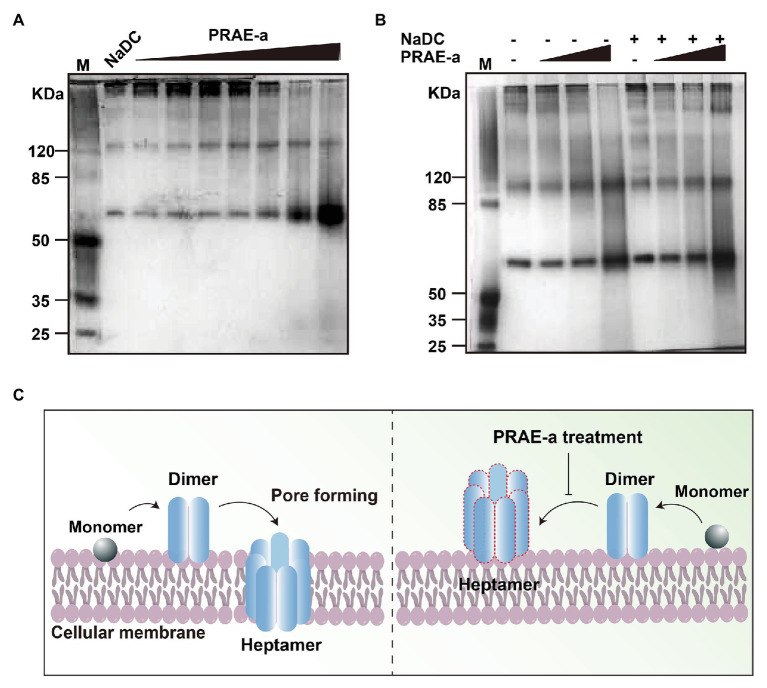
PRAE-a inhibited the hemolytic activity of α-toxin by destroying the α-toxin to form dimerization. **(A)** PRAE-a promoted the dimerization in a dose-dependent manner. M represents the marker. PRAE-a solution was incubated for 30 min with α-toxin (50 μg/ml) at concentrations of 0, 0.06, 0.15, 0.25, 0.5, 1.0, and 2.0 mg/ml. The result of native polyacrylamide gel electrophoresis showed the protein bands of α-toxin. NaDC was used as a control, which usually promotes α-toxin to form oligomer. **(B)** PRAE-a interrupted the process of oligomer mediated by NaDC. PRAE-a solution (0.15, 0.5, and 2 mg/ml) were incubated with α-toxin for 30 min in the presence or absence NaDC. The native polyacrylamide gel electrophoresis analysis showed the bands of the proteins of α-toxin. **(C)** The scheme of PRAE-a inhibited the hemolytic activity of α-toxin.

Previous studies revealed that the hemolytic toxin monomer needed to assemble into heptamer on the target cell membrane to achieve perforation damage (von Hoven et al., 2019). To further investigate how PRAE-a abolished the hemolytic activity of α-toxin, we used native polyacrylamide gel electrophoresis to detect the assembly of α-toxin under PRAE-a treatment at a protein level. The protective agent of endotoxin, sodium deoxycholate (NaDC), was employed as positive control to promote the self-assembly of α-toxin monomer into oligomers. We found that administration of NaDC alone promoted that polymerization of hemolytic toxin monomers to form 2, 4, 6, and 7-oligomer. However, when induced by additional PRAE-a, it was observed that with increasing PRAE-a concentration, the formation of dimers was significantly increased, but the formation of high oligomers was significantly decreased with a significant dose dependence (1 or 2 mg/ml, Figure 6A). In addition, the hemolytic toxin had a tendency to self-assemble without the addition of NaDC, but the ability to self-assemble into high oligomers was markedly decreased with the addition of PRAE (Figure 6B). These results illustrated that the mechanism of PRAE-a abolished the hemolytic activity of α-toxin by destroying α-toxin to lead to accumulation of α-toxin dimer, which is useless for pore forming of α-toxin on the cellular membrane (Figure 6C).

### PRAE-a Therapy of *S. aureus* Lung Infection by Reducing α-Toxin

**Figure 7:**
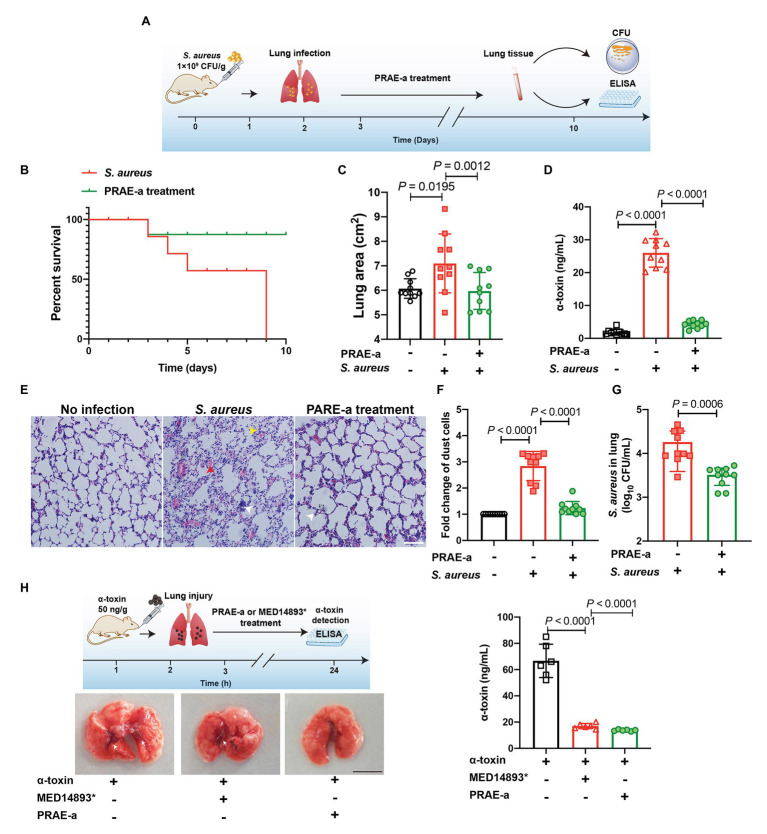
PRAE-a suppressed infection of *S. aureus* α-toxin in the mice. **(A)** Lung infection by *S. aureus* and PRAE-a treatment workflow. **(B)** PRAE-a increased the survival of mice that were infected by *S. aureus* under α-toxin addition. Survival of mice that were infected by *S. aureus* (10^9^ CFU/g, blue line) or *S. aureus* addition were determined. PRAE-a (1 mg/g bw) treatment (green line) was carried out in the group of *S. aureus* with the addition of extra α-toxin. **(C)** The lung areas were determined and calculated by image J software. **(D)** The concentrations of α-toxin in the lung were detected by ELISA. **(E)** PRAE-a prevented the inflammatory cell infiltration induced by α-toxin. Numbers of dust cells were counted from D. **(F)** H&E staining of the lung tissue. Red arrowheads referred to tissue necrosis, white arrowheads represented inflammatory cell infiltration, and yellow arrowheads showed the alveolar rupture. **(G)** The extract PRAE-A decreased *S. aureus* expansion in the lung. The number of *S. aureus* in the lung of the infected mouse were detected by colony count technique (CFUs). **(H)** PRAE-a decreased the lung injury of α-toxin by inhibiting the levels of α-toxin in the lung. Mice were pretreated with α-toxin (50 ng/g) orally. After 3 h, the mice were then treated with PRAE-a or MED14893*. Finally, the concentration of α-toxin in lung tissue was detected by ELISA. The experiment workflow was shown on the upper left of the figure and the images of the lung were captured and the bleeding spots were marked by white arrowheads. The scale bar was 1 cm. The levels of α-toxin in each lung, which was shown on the right of the figure, were detected as shown in previous methods by ELISA.

We next sought to evaluate the therapy of PRAE-a for *S. aureus* infection. A pulmonary infection model in mice demonstrated that α-toxin facilitated the death of *S. aureus*-infected mice (Figure 7A), whereas PRAE-a treatment increased the survival of the infected mice (Figure 7B). In addition, PRAE-a could effectively ameliorate lung enlargement in the infected mice (Figure 7C). The concentration of α-toxin in the lung decreased after PRAE-a treatment (Figure 7D), which was consistent with the result that PRAE-a directly inhibited α-toxin (Figure 3). In addition, PRAE-a prevented mice from developing tissue necrosis, inflammatory cell infiltration, and alveolar rupture in lung caused by α-toxin of *S. aureus* in the infected mice (Figure 7E). And PRAE-a might have an anti-inflammatory effect by decreasing α-toxin-induced dust cell increase (Figure 7F). We also found that PRAE-a effectively reduced the numbers of *S. aureus* in the lung (Figure 7G). Results suggested that the bacterial loading in the lung were controlled by PRAE-a treatment and it was tightly associated with the direct inhibition of α-toxin by PRAE-a, because α-toxin could promote bacterial lung infection (Cohen et al., 2016). To further investigate the injury of the lung caused by α-toxin, mice were pretreated with α-toxin orally and the therapeutic effect of PRAE-a was evaluated; we found that both PRAE-a and positive control MED14893^*^ could inhibit the levels of α-toxin in the lung, but only PRAE-a decreased the lung injury of α-toxin (Figure 7D). Taken together, PRAE-a could be used as a drug candidate for the therapy of the α-toxin-caused pulmonary *S. aureus* infection.

## Discussion

In recent decades, efforts have been made to develop antibacterial drugs (Ling et al., 2015; Taccone et al., 2016; Song et al., 2020b). However, drug discovery is difficult due to the emerging of drug resistance (Brown, 2015; Bonten, 2016). In addition, infections caused by tolerant and persistent bacteria are worldwide (Grant and Hung, 2013; Becattini et al., 2016). Bacteria exposed to antibiotics at their sublethal levels are enough to lead to the formation of antibiotic tolerance and, in this process, antibiotics in turn promote the production of bacterial toxins (Liu et al., 2020b). Most recently, invasion of pathogenic bacteria and inflammation caused by toxins has been frequently emerging (Czuczman et al., 2014; Mohamed et al., 2014; Fox et al., 2020). Moreover, the relapse and recurrence of infections caused by bacterial toxins are becoming uncontrollable (Vadia et al., 2011; Cohen et al., 2016). Therefore, more efforts are needed for drug discovery.

Natural herbs are increasingly being shown to have antitoxic activity. For example, Lysionotin targeted inhibition of α-toxin expression to decrease the pathogenicity of *S. aureus* (Teng et al., 2017) and Chalcone reduced the virulence of *S. aureus* by targeting sortase A and α-toxin (Zhang et al., 2017). These reports were consistent with our finding that PRAE-a have significant antihemolytic activity against *S. aureus* α-toxin (Figure 2). Additionally, the fact that the compound of PRAE-a was a paeoniflorin - like structure had been determined (Figures 3–5). Interestingly, the dry standard drug paeoniflorin did not have as significant antihemolytic activity (Figures 2B,D), which suggested that the formation process of traditional Chinese herbs is critical for maintaining the activity of herbs (Dutkiewicz et al., 2001; Li et al., 2019). Subsequently, to further illustrate the mechanisms of how PRAE-a targeted *S. aureus* α-toxin, we found that the interaction between PRAE-a and α-toxin had a direct impact on interrupting α-toxin to form heptamer (Figures 6B–C), which further abolished the pore forming ability of α-toxin on cellular membrane. It was similar to the action of Aloe-emodin and Baicalin that directly interfered with the oligomerization of α-toxin to attenuate *S. aureus* pathogenicity (Qiu et al., 2012; Jiang et al., 2019).

Overall, these functional mechanisms of PRAE-a reminded us of developing the medicinal value of PRAE-a for therapy against infectious disease. Therefore, we constructed a mouse model of pulmonary infection to evaluate the treatment effect of PRAE-a (Figure 7A) and results showed PRAE-a inhibited the bacterial infection and decreased the level of α-toxin in the lung (Figures 7B–G). In our previous work, we found PRAE-a did not have a good antibacterial effect because of the MIC of PRAE-a to *S. aureus* was 6.3 mg/ml. However, the concentrations of PRAE-a that we used in this study were much less than the MIC and it still showed antihemolytic activity (Figure 2). Therefore, we thought it had few effects on bacteria themselves in this concentration and it directly acted on α-toxin as shown in Figure 6. However, we indeed found that PRAE-a could decrease the bacterial loading in the lung *in vivo* (Figure 7G). It was also associated with the inhibition of α-toxin by PRAE-a (Figure 7D) as *S. aureus* α-toxin can increase the bacterial invasion *in vivo* (Maurer et al., 2015; Cohen et al., 2016). In addition, we further found that α-toxin damaged the lung tissue and PRAE-a can rescue the injury caused by α-toxin (Figure 7H). Taken together, the results revealed that PRAE-a altered the death rate caused by *S. aureus* α-toxin and decreased the amount of *S. aureus* in the lung of the infected mouse (Figures 7B,D,G), which was directly related to the decrease of the concentrations of α-toxin in the lung (Figure 7F). Thus, we believe that our work on the antitoxic function of PRAE-a will highlight the potential use of traditional Chinese natural herbs for new antimicrobial strategies that target bacterial toxins.

## Data Availability Statement

All experimental raw data supporting the conclusions of this article will be made available by the authors, without undue reservation.

## Ethics Statement

All experimental animal procedures were reviewed and approved by the Institutional Animal Care and Use Committee at the Academy of Military Medical Sciences Institute (Beijing, China; approval no. SYXK2014-0002).

## Author Contributions

Y-JS and Y-JW conceived of the project. ZL, PZ, and YZ did the experiments. XL, ZL, and PZ performed the data analysis. ZL, XL, Y-JS, and Y-JW wrote the paper. All authors contributed to the article and approved the submitted version.

### Conflict of Interest

The authors declare that the research was conducted in the absence of any commercial or financial relationships that could be construed as a potential conflict of interest.

## Footnotes

^1^http://www.weipujishu.com/
